# Dislocation of the Sternal End of the Clavicle, Upwards and Backwards

**Published:** 1884-11

**Authors:** 


### Dislocation of the Sternal End of the Clavicle, Upwards and Backwards.

J. Harry Poland, in the *Lancet* of July 19th, reports a case of this comparatively rare luxation. The patient, a young man, aged 20, was admitted into the Sandhurst Hospital, Victoria, with the history of a fall while wrestling, he being underneath. He complained of pain in the right shoulder, and inability to use the right arm, slight difficulty in breathing, and great difficulty and pain in swallowing. On examination, the right shoulder was slightly lower than the left, and projected more forwards; the arm was extended and kept at an angle of 40° to the body, this being the least painful position. The head was inclined and turned slightly to the left side. The line of the clavicle could be felt throughout. There was a well-marked depression at the right sterno-clavicular articulation, and the sternal end of the clavicle could be felt beyond this, and at about three-quarters of an inch higher level than the corresponding articulation, the trachea apparently being pushed to the left side. The patient was seated in a chair, and reduction effected by placing the knee in the hollow between the two scapulæ, taking firm hold of the upper part of the arm and shoulder, and bringing them backwards and a little upwards. The parts were retained in position by means of a large pad placed over the right scapula, and the right arm bandaged so as to keep it well backwards, the elbow a little upwards and the fore-arm folded across the chest. There was some difficulty in keeping the arms in position, and the bandage had to be reapplied three or four times. The head of the clavicle did not completely resume its former position, and at the end of four weeks, when the patient was discharged, a slight depression could be felt over the articulation. There was, however, not the slightest trace of any upward displacement. The patient has since resumed work, and experiences no inconvenience from the injury.

